# *BoLA-DRB3* Polymorphism Controls Proviral Load and Infectivity of Bovine Leukemia Virus (BLV) in Milk

**DOI:** 10.3390/pathogens11020210

**Published:** 2022-02-05

**Authors:** Ayumi Nakatsuchi, Sonoko Watanuki, Liushiqi Borjigin, Hirotaka Sato, Lanlan Bai, Ryosuke Matsuura, Maho Kuroda, Hironobu Murakami, Reiichiro Sato, Sakurako Asaji, Asako Ando, Yasunobu Matsumoto, Shin-Nosuke Takeshima, Yoko Aida

**Affiliations:** 1 Institute of Animal Health, JA Zen-Noh (National Federation of Agricultural Cooperative Associations), 7 Ohja-machi Sakura-shi, Chiba 285-0043, Japan; nakatsuchi-ayumi@zennoh.or.jp (A.N.); kuroda-maho-z@zennoh.or.jp (M.K.); 2 Laboratory of Global Animal Resource Science, Graduate School of Agricultural and Life Sciences, The University of Tokyo, 1-1-1 Yayoi, Bunkyo-ku, Tokyo 113-8657, Japan; watanuki.sonoko917@gmail.com (S.W.); matsumoto-yasu@mvi.biglobe.ne.jp (Y.M.); 3 Laboratory of Global Infectious Diseases Control Science, Graduate School of Agricultural and Life Sciences, The University of Tokyo, 1-1-1 Yayoi, Bunkyo-ku, Tokyo 113-8657, Japan; matsuura-ryosuke@g.ecc.u-tokyo.ac.jp; 4 Viral Infectious Diseases Unit, RIKEN, 2-1 Hirosawa, Wako, Saitama 351-0198, Japan; liushiqi.borjigin@vetmed.hokudai.ac.jp (L.B.); hirosato@dokkyomed.ac.jp (H.S.); lanlan.bai@riken.jp (L.B.); takesima@jumonji-u.ac.jp (S.-N.T.); 5 Photonics Control Technology Team, RIKEN Center for Advanced Photonics, 2-1 Hirosawa, Wako, Saitama 351-0198, Japan; 6 Laboratory of Animal Health II, School of Veterinary Medicine, Azabu University, 1-17-71 Fuchinobe, Chuo-ku, Kanagawa, Sagamihara 252-5201, Japan; h-murakami@azabu-u.ac.jp; 7 Laboratory of Farm Animal Internal Medicine, Department of Veterinary Sciences, Faculty of Agriculture, University of Miyazaki, Miyazaki 889-2192, Japan; r-sato@cc.miyazaki-u.ac.jp; 8 GenoDive Pharma Inc., 4-14-1 Naka-cho, Kanagawa, Atsugi 243-0018, Japan; sasaji@genodive.co.jp (S.A.); aando@is.icc.u-tokai.ac.jp (A.A.); 9 Department of Molecular Life Science, Division of Basic Medical Science and Molecular Medicine, Tokai University School of Medicine, 143 Shimokasuya, Kanagasa, Isehara 259-1193, Japan; 10 Department of Food and Nutrition, Jumonji University, 2-1-28 Sugasawa, Niiza, Saitama 352-8510, Japan

**Keywords:** bovine leukemia virus (BLV), *BoLA-DRB3* allele, milk, dam, proviral load, infectivity, vertical transmission, visualization, susceptible, resistant

## Abstract

Bovine leukemia virus (BLV), which causes enzootic bovine leukosis, is transmitted to calves through the milk of BLV-infected dams. Bovine leukocyte antigen (BoLA)-*DRB3* is a polymorphic gene associated with BLV infectivity and proviral load (PVL). However, the effect of *BoLA-DRB3* polymorphism on the infectivity and PVL of milk from BLV-infected dams remains unknown. This study examined milk from 259 BLV-infected dams, including susceptible dams carrying at least one BoLA-DRB3*012:01 or *015:01 allele with high PVL, resistant dams carrying at least one BoLA-DRB3*002:01, *009:02, or *014:01:01 allele with low PVL, and neutral dams carrying other alleles. The detection rate of BLV provirus and PVL were significantly higher in milk from susceptible dams than in that from resistant dams. This result was confirmed in a three-year follow-up study in which milk from susceptible dams showed a higher BLV provirus detection rate over a longer period than that from resistant dams. The visualization of infectivity of milk cells using a luminescence syncytium induction assay showed that the infectious risk of milk from BLV-infected dams was markedly high for susceptible dams compared to resistant ones. This is the first report confirming that *BoLA-DRB3* polymorphism affects the PVL and infectivity of milk from BLV-infected dams.

## 1. Introduction

Bovine leukemia virus (BLV) belongs to the genus *Deltaretrovirus* of the family *Retroviridae*, which also includes human T-cell leukemia virus types 1 and 2 (HTLV-1 and -2). BLV causes enzootic bovine leukosis (EBL), the most common neoplastic disease affecting cattle worldwide [1]. BLV is integrated as a provirus into the DNA of B lymphocytes and a wide range of cell types, and infection lasts a lifetime. Approximately 70% of cattle infected with BLV are asymptomatic, but approximately 30% develop symptoms of persistent lymphocytosis and 1–5% develop malignant lymphoma [1]. BLV infections have spread worldwide and affect an estimated 40.9% of dairy cattle in Japan [2]. Economic damage associated with BLV infection includes low meat and milk production, and shorter productive life due to BLV infection, and damage associated with the discarding of cattle due to lymphoma [3,4,5,6]. In addition, BLV infection affects the innate and adaptive immune systems, altering the normal function of uninfected cells and resulting in increased susceptibility to infection and disease severity in BLV-infected cattle [7,8,9].

BLV is transmitted primarily through the transfer of infected lymphocytes via horizontal and vertical routes [10]. Horizontal transmission, which refers to the spread of BLV through blood containing infected cells, can occur through repeated use of syringes and gloves for rectal examinations, dehorning, and castration as well as through blood-sucking insects [11,12,13]. Vertical transmission occurs via dam–calf contact through intrauterine infection of the fetus and/or ingestion of milk and colostrum from BLV-infected dams [14,15,16,17,18]. We previously reported that BLV-infected cells are infectious in milk [19]. Moreover, a previous study reported that lambs became infected with BLV after inoculation with milk or viable milk cells from BLV-infected dairy cattle [20]. In addition, HTLV-1, a virus related to BLV, is transmitted through milk, and determination of the transmission mechanism of BLV through milk should be applied to the study of vertical transmission of HTLV-1 [21]. These findings suggest that controlling the ingestion of infected colostrum and milk may reduce vertical transmission of BLV on farms.

Major histocompatibility complex (MHC) genes encode glycoproteins on the surface of cell membranes involved in the immune response. MHC genes are associated with disease susceptibility because of their ability to present various intracellular peptides on the cell surface [22]. The gene encoding MHC in cattle, bovine leukocyte antigen (BoLA), contains a highly polymorphic BoLA class II locus at chromosome 23. A total of 384 *BoLA-DRB3* alleles are registered in the Immuno-Polymorphism Database (IPD)-MHC database (https://www.ebi.ac.uk/ipd/mhc/group/BoLA/) (accessed on 28 October 2021). Previous studies have shown that *BoLA-DRB3* polymorphism in BLV-infected cattle is related to blood proviral load (PVL), infectivity, lymphoma, and in utero infection of calves [18,23,24,25,26,27,28,29]. In addition to BLV, *BoLA-DRB3* polymorphism is associated with infectious diseases such as bovine clinical mastitis, papillomavirus infection, herpes virus type 1 infection, and foot-and-mouth disease [30,31,32,33]. Therefore, the *BoLA-DRB3* polymorphism is considered a useful gene in immunology. However, the effect of *BoLA-DRB3* polymorphism on milk from dams infected with BLV is unclear.

Our previous study demonstrated that the PVL in milk was positively associated with that in blood; BLV provirus was detected in milk samples when blood PVL was above approximately 10,000 copies/10^5^ cells and milk cells were infectious ex vivo [19]. However, we have not examined the effect of *BoLA-DRB3* polymorphism on the milk from BLV-infected cattle. In this study, we evaluated the effect of *BoLA-DRB3* polymorphism on PVL in the milk of BLV-infected cattle using BLV-CoCoMo-quantitative-Polymerase-Chain-Reaction (qPCR)-2, an assay developed for the sensitive detection of BLV at one point and a three-year follow-up investigation. Similarly, we evaluated the effect of *BoLA-DRB3* polymorphism on the infectivity of milk cells using an improved luminescence syncytium induction assay (LuSIA), optimized for milk cells and enabling visualization of BLV cell-to-cell infection in vitro.

## 2. Results

### 2.1. BLV Infection Rate and Frequencies of Susceptible, Neutral, and Resistant Dams on Different Dairy Farms

To investigate the effect of *BoLA-DRB3* polymorphism on milk from BLV-infected dams, we first collected peripheral blood from 672 Holstein-Friesian cattle on eight dairy farms throughout Japan to determine the presence of BLV infection (Table 1). DNA was extracted from blood samples to measure the PVL of known and novel BLV variants in infected cattle using the BLV-CoCoMo-qPCR-2 assay (RIKEN Genesis, Kanagawa, Japan) [34,35,36,37]. In total, 300 of 672 cattle (44.6%) were positive for BLV PVL, and the BLV-positivity rate ranged from 14.7 to 71.7% among the farms (Table 1).

Among the 536 dams, 259 (48.3%) were positive for BLV PVL, and the BLV-positivity rate ranged from 16.6 to 78.5% among the farms (Table 1). The average PVL in blood ranged from 12,440 to 30,066 copies/10^5^ cells, with an average of 22,080 copies/10^5^ cells across the eight dairy farms. Furthermore, PCR-sequence-based typing (SBT) identified 12 known *BoLA-DRB3* alleles in the IPD-MHC database that were used to divide the dams into susceptible, resistant, and neutral groups. Susceptible dams carried at least one susceptible BoLA-DRB3*012:01 or *015:01 allele and were associated with high BLV PVL. Resistant dams carried at least one resistant BoLA-DRB3*002:01, *009:02, or *014:01:01 allele and were associated with low BLV PVL [23,25,38,39]. Dams carrying both resistance and susceptible alleles were defined as resistant because the resistance trait was more dominant than the susceptibility trait [28]. Neutral dams carried other alleles. The rate of susceptible, neutral, and resistant dams ranged from 22.5 to 47.2% (mean 35.3%), 26.4 to 60.0% (mean 43.8%), and 7.6 to 33.3% (mean 20.9%), respectively, among the farms (Table 1).

### 2.2. BLV Provirus Detection Rate and PVL in Milk from Susceptible, Neutral, and Resistant Dams Infected with BLV

**Figure 1:**
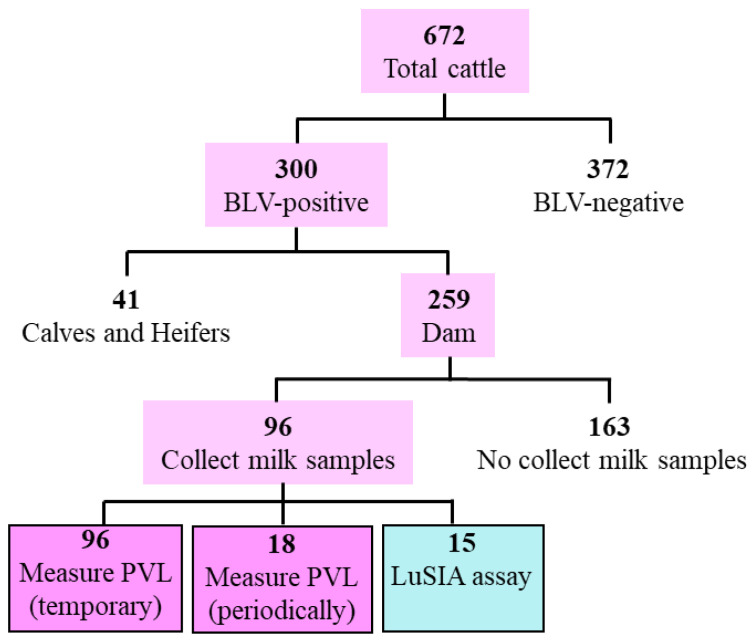
Flow diagram of sample selection. Cattle blood DNA samples were subjected to the CoCoMo-qPCR-2 assay, following which 672 cattle were diagnosed with bovine leukemia virus (BLV) infection, among which 300 cattle were BLV-positive, including 259 dams. BLV proviral load (PVL) was temporary measured in milk samples from 96 of 259 BLV-positive dams. In addition, 18 of 96 dams were periodically assessed for BLV PVL in milk over three years, and milk samples from 15 of 96 dams were used to optimize luminescence syncytium induction assay (LuSIA) for milk cells.

**Figure 2:**
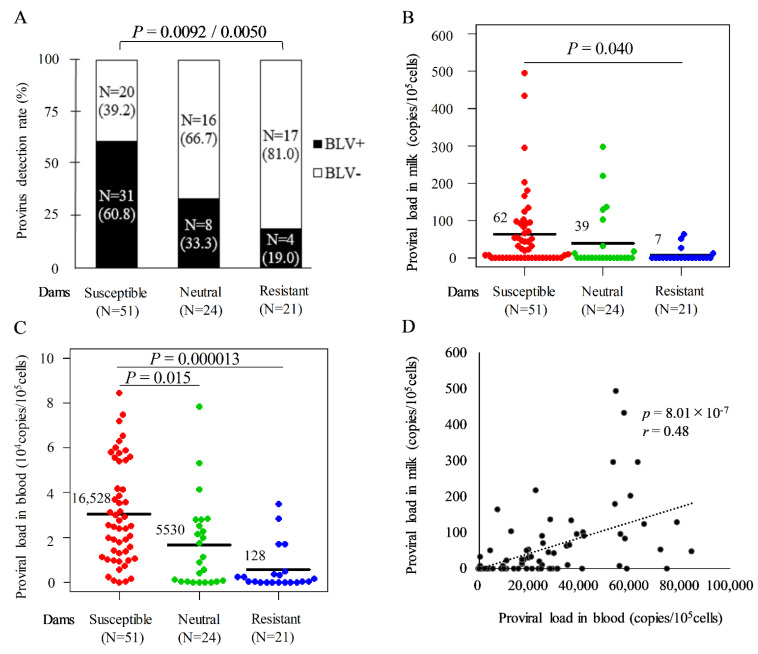
Positivity rates for bovine leukemia virus (BLV) provirus and estimation of proviral load (PVL) in milk from susceptible, resistant, and neutral dams. Milk and blood samples were obtained from 96 BLV-positive dams and extracted DNAs. The PVLs in milk and blood were measured using the CoCoMo-qPCR-2 method (RIKEN Genesis, Kanagawa, Japan), DNA from milk and blood, and *BoLA-DRB3* alleles were typed by the PCR-SBT method using DNA from blood. All BLV-positive dams divided into resistant, susceptible, and neutral groups based on the presence of *BoLA-DRB3* alleles, as follows: susceptible dams carried at least one BoLA-DRB3*012:01 or *015:01 allele in their genomes; resistant dams carried at least one BoLA-DRB3*002:01, *009:02, or *014:01:01 allele in their genomes; and neutral dams carried other alleles in their genomes. Dams carrying both susceptible and resistant alleles were defined as resistant. (**A**) Effect of *BoLA-DRB3* polymorphism on BLV provirus detection rate in milk from BLV-infected dams. *p-*values were adjusted using the Benjamini & Hochberg method and derived using the chi-square test (*p* = 0.0092) and Fisher’s exact test (*p* = 0.0050). (**B**,**C**) Comparison of PVL in milk and blood from BLV-infected dams. Mean PVL values were compared among groups using the Tukey’s multiple comparison test. (**D**) Correlation between PVL in milk (from (**B**)) and blood (from (**C**)). The bold line represents the approximate curve (*r* = correlation coefficient), and the *p*-value was derived using the Pearson function (*p* = 8.01 × 10^−7^).

Previously, we detected BLV provirus in milk samples from 22 of 48 cattle using the BLV-CoCoMo-qPCR-2 assay [19]. To investigate whether *BoLA-DRB3* polymorphism affected the detection rate of BLV provirus in milk, we collected milk samples from 96 of 259 BLV-infected dams (Figure 1), excluding dams that could not be milked because of a dry period, mastitis, or farmer inconvenience at the time of testing. Furthermore, BLV provirus was detected in milk from 31 of 51 susceptible, eight of 24 neutral, and four of 21 resistant dams (Figure 2A). The rate of BLV provirus detection differed significantly (*p* < 0.05) between susceptible and resistant groups using the chi-square test and Fisher’s exact test. Our results revealed that the BLV provirus detection rate in milk from susceptible dams was significantly higher than that from resistant dams.

Next, we evaluated whether *BoLA-DRB3* polymorphism affected the BLV PVL in milk as well as the BLV provirus detection rate (Figure 2B). The average PVL values in milk were 62, 39, and 7 copies/10^5^ cells for the susceptible, neutral, and resistant groups, respectively. Moreover, the average PVL in milk from the susceptible group was significantly higher than that from the resistant group (*p* = 0.040). Furthermore, mean PVL values in blood were 16,528, 5530, and 128 copies/10^5^ cells for the susceptible, neutral, and resistant groups, respectively (Figure 2C). These results indicated that the PVL in blood of susceptible dams was significantly higher than that of neutral (*p* = 0.015) and resistant dams (*p* = 0.000013), as well as that in milk (Figure 2B,C). Figure 2D shows that the correlation coefficient (*r*) was 0.48 (*p* = 8.01 × 10^−7^), indicating that although the PVL in milk was lower than that in blood, a positive correlation existed between the PVL in milk and blood from BLV-infected dams.

### 2.3. Genotype of BoLA-DRB3 Allele Is Associated with BLV PVL in Milk

**Figure 3:**
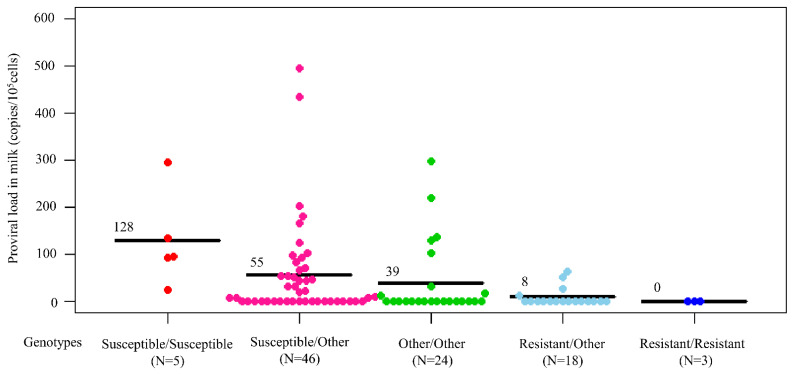
Bovine leukemia virus (BLV) proviral load (PVL) in milk from 96 BLV-infected dams with different *BoLA-DRB3* genotypes including susceptible/susceptible allele, susceptible/other allele, other/other allele, resistant/other allele, and resistant/resistant allele genotypes. Mean PVL values among groups were compared using Tukey’s multiple comparison test.

Next, the BLV-positive dams were divided into five groups based on whether their genotypes were homozygous or heterozygous for the susceptible and resistant alleles. PVL in milk was compared based on each genotype (Figure 3). The results indicated that five dams carried a homozygous genotype for susceptible alleles had the highest mean BLV PVL in milk (128 copies/10^5^ cells), followed by 46 dams with a heterozygous genotype for a susceptible allele with another allele (55 copies/10^5^ cells), 24 dams with a homozygous genotype for another allele (39 copies/10^5^ cells), 18 dams with a heterozygous genotype for a resistant allele with another allele or susceptible allele (8 copies/10^5^ cells), and three dams with a homozygous genotype for a resistant allele (0 copies/10^5^ cells). Thus, BLV PVL was the highest in milk from dams possessing a homozygous genotype for susceptible alleles, while it was the lowest in milk from dams possessing a homozygous genotype for resistant alleles.

### 2.4. Assessing BLV PVL in Milk from Susceptible and Resistant Dams Infected with BLV in a Longitudinal Follow-Up Study

The experimental results indicated that *BoLA-DRB3* alleles affected BLV provirus detection and the PVL in milk, particularly in susceptible and resistant dams. To confirm whether these effects were continuous rather than temporary, 18 BLV-infected dams were selected from farms A and E among the 259 BLV-infected dams, including 13 susceptible (S1 to S13) and five resistant dams (R1 to R5) (Table 2). The PVL in milk samples collected from these dams was periodically tested seven times over three years from July 2017 to August 2019 (Table 2), except when milk samples could not be obtained because of a dry period, mastitis, or farmer’s convenience at the time of sampling. The results of the three-year follow-up analysis revealed that the frequency of BLV provirus detection in milk from susceptible dams was consistently higher than that from resistant dams. BLV provirus was continuously undetectable in the milk from four of five (R2 to R5) resistant dams (80%) at all testing points during the follow-up period. In contrast, only milk from 1 of 13 susceptible dams (8%) (S13 with low PVL of 6389 copies/10^5^ cells (October 2017) in blood) was negative for BLV provirus at all four testing points during the follow-up period. In addition, BLV provirus was detected in milk from two of 13 susceptible dams (S1 and S2 with high PVL of 63,280 (July 2017) and 49,799 copies/10^5^ cells (October 2017) in blood, respectively) at all testing points and in milk from 10 of 13 susceptible dams at two to four testing points during the follow-up period. Our findings demonstrated that milk from resistant dams had low PVL during the long infectious period compared to that of susceptible dams.

### 2.5. Evaluation of Infectivity of Milk Cells from Susceptible and Resistant Dams Infected with BLV

**Figure 4:**
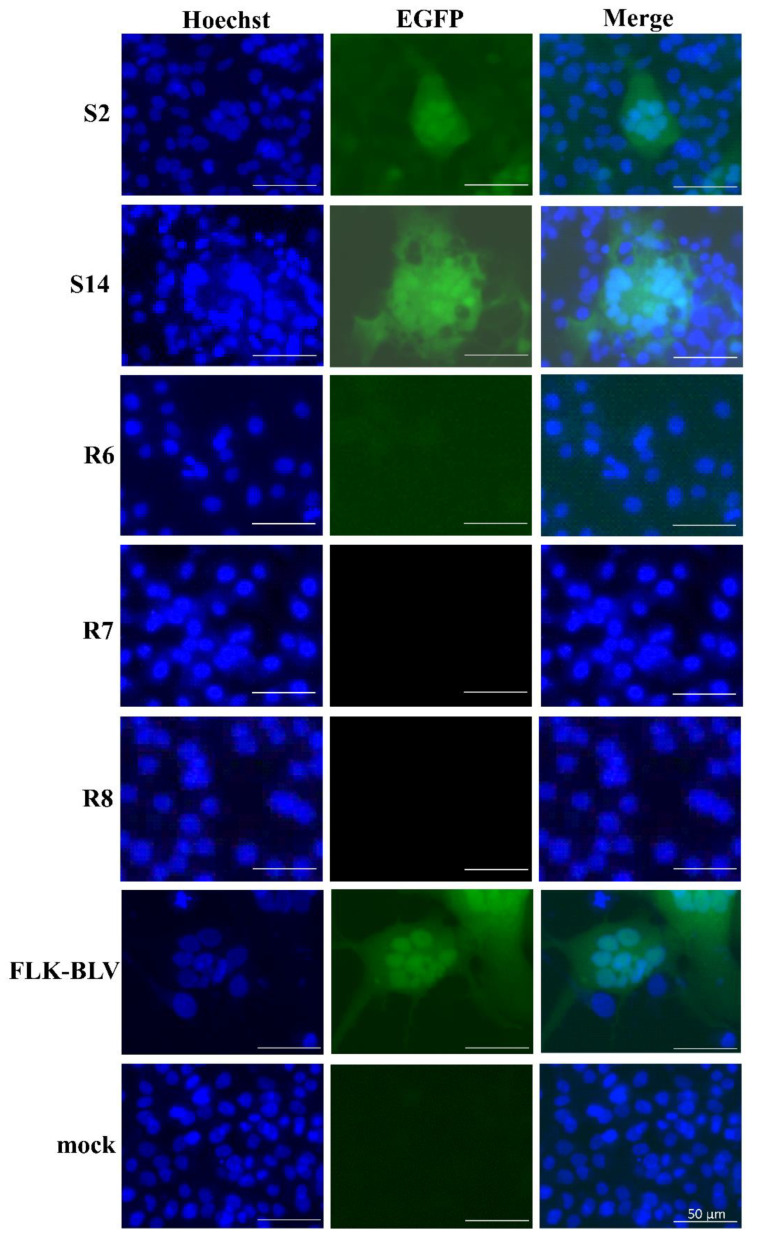
Representative visualization of the infectivity of milk cells from bovine leukemia virus (BLV)-positive dams (S2 and S14 susceptible dam; R6, R7 and R8 resistant dam) by an improved luminescence syncytium induction assay (LuSIA). Enhanced green fluorescent protein (EGFP)-fluorescent syncytia were observed using an EVOS2 fluorescence microscope or a BZ-X810 fluorescence microscope. The merged image is composed of overlaid Hoechst and EGFP images. FLK-BLV cells, which were productively infected with BLV, were used as the positive control. In the mock image, CC81-GREMG cells were cultured without milk cells. Scale bars = 50 µm.

Our previous study developed a LuSIA based on the new CC81 (a feline cell line transformed by mouse sarcoma virus)-derived reporter cell line, CC81-GREMG [40,41]. This cell line specifically responds to the expression of the BLV regulatory protein, Tax, when cultured together with BLV-infected cells, forming enhanced green fluorescent protein (EGFP)-expressing syncytia. This assay was further optimized for milk cells, determining the BLV infectivity of milk cells in samples collected from seven BLV-infected dams [19]. To clarify whether *BoLA-DRB3* polymorphism affected the infectivity of BLV in milk cells, nine susceptible and six resistant dams raised at farms A, C, D, and E were selected from the 259 BLV-infected dams, and BLV infectivity in milk was assessed using improved LuSIA (Table 3). Milk cells were isolated from the milk of BLV-infected dams and co-cultured with CC81-GREMG with pokeweed mitogen (PWM) for five days. Nuclei were stained with Hoechst 33342, which showed blue fluorescence. EGFP-expressing syncytia were observed using an EVOS2 fluorescence microscope and a BZ-X810 fluorescence microscope. Figure 4 displays representative images of milk cells from two BLV-positive susceptible dams S2 with a PVL of 70 copies/10^5^ cells in milk and S14 with a PVL of 107 copies/10^5^ cells in milk, showing a large syncytium expressing EGFP, compared to milk cells of BLV-positive resistant dams (R6, R7 and R8) and permanently BLV-infected FLK-BLV cells. In addition to those from S2 and S14, milk cells from three of nine susceptible dams (S15, S16, and S20 with PVL values of 66, 56, and 30 copies/10^5^ cells in milk, respectively) were considered infectious based on the LuSIA results (Table 3). In contrast, milk cells from all six resistant dams, including R6 and R7 with PVL values of 108 and 51 copies/10^5^ cells in milk, respectively, were considered non-infectious based on the LuSIA results. These results indicated that the infectious risk of milk from BLV-infected dams was significantly higher for dams with susceptible alleles than for those with resistant alleles.

## 3. Discussion

Our previous study demonstrated that milk cells from BLV-infected dams are infectious ex vivo [19]. In addition, lambs became infected with BLV after being inoculated with colostrum and milk obtained from BLV-infected cattle [20]. These findings suggest that milk is a potential risk factor for the spread of BLV through cell-to-cell transmission. The current study evaluated the BLV transmission risk of milk from dams harboring different *BoLA-DRB3* alleles, demonstrating for the first time that *BoLA-DRB3* polymorphism affects the frequency of BLV provirus detection, PVL, and ex vivo infectivity. First, the frequency of BLV provirus detection and BLV PVL in milk from susceptible dams was significantly higher than that of resistant dams. The order of these outcomes was as follows: susceptible > neutral > resistant dams. Additionally, the effects of *BoLA-DRB3* polymorphism were observed over a long period; BLV PVL in milk of susceptible dams was continuously detectable over three years but less detectable in milk of resistant dams. Second, milk cells from five of nine susceptible dams were infectious, while those from all six resistant dams were non-infectious. Thus, our results confirm that milk from susceptible BLV-infected dams has a higher risk of vertical transmission than that from resistant dams. Similarly, a previous study reported that perinatal transmission risk was markedly low for dams and calves carrying resistant alleles compared to those harboring susceptible alleles [18]. In addition, BLV-infected susceptible cattle were at a higher risk of horizontal transmission risk was reportedly high in BLV-infected susceptible dams with high infection severity and high PVL in blood compared to those with low infection severity and low PVL in blood [26]. Thus, vertical and horizontal transmission risk is higher for dams carrying susceptible alleles than those harboring resistant alleles, suggesting that BLV infection may be controlled using measures guided by *BoLA-DRB3* polymorphism. Third, dams possessing homozygous genotypes for susceptible alleles had the highest BLV PVL in milk compared to those possessing other genotypes. These findings provide the first evidence that susceptible alleles identified in blood are risk indicators for persistent BLV provirus detection and BLV PVL in milk, supporting a previous study, which reported that *BoLA-DRB3* homozygotes have a disadvantage against BLV-induced lymphoma and PVL in blood [24].

The present study revealed significant differences in BLV PVL in milk from susceptible and resistant dams, which concurred with the results of a previous study in which PVL in blood was ranked in order of susceptible, neutral, and resistant cattle [26]. Furthermore, our previous study determined a positive correlation between PVL in blood and milk from BLV-infected dams, and identical partial BLV envelope gene (*env*) sequences in milk and blood from the same dam indicated that the BLV detected in milk was of blood origin [19]. However, BLV *env* sequencing was not performed in this study, and based on our previous report, we consider that PVL in milk is always of blood origin [19]. Moreover, previous studies have demonstrated that *BoLA-DRB3* polymorphism is useful in risk assessment of BLV-infected cattle because it is associated with BLV PVL in blood and in utero infection [18,23,26]. Therefore, the PVL in milk might be equally useful for risk assessment of BLV vertical transmission through milk.

Further dividing susceptible and resistant dams based on homozygous or heterozygous genotypes for susceptible or resistant alleles revealed that dams with homozygous susceptible genotypes had the highest average PVL in milk, while dams possessing homozygous resistant genotypes had the lowest average PVL in milk. A previous study reported that BLV PVL in blood of cattle with homozygous susceptible genotypes was significantly higher than that in cattle with heterozygous resistant/neutral genotypes [18]. However, the study did not evaluate BLV PVL in the blood of cattle with homozygous resistant genotypes. To the best of our knowledge, the present study is the first to report that homozygous susceptible and resistant genotypes are associated with different effects on BLV PVL in milk. Moreover, Lo et al. reported that the BLV PVL in blood of Holstein-Friesian cattle possessing homozygous genotypes, regardless of allele type, was significantly higher than that in blood of cattle with heterozygous genotypes, and the frequency of development of lymphoma was increased in the former [24]. Therefore, it is important to consider not only the presence of susceptible or resistant alleles but also the genotype of *BoLA-DRB3* when assessing the effect of *BoLA-DRB3* polymorphism on BLV infection. Regardless, further studies with more dams are required to confirm the effect of genotype on the PVL in milk from BLV-infected cattle.

The current study demonstrated that *BoLA-DRB3* polymorphism affects BLV provirus detection and BLV PVL in milk, and this effect occurs continuously rather than transiently. Moreover, BLV provirus was detected more frequently and continuously in milk from dams, especially susceptible dams, with a PVL greater than 10,000 copies/10^5^ cells in blood during the three-year follow-up study. Our findings concur with a previous study reporting that BLV provirus was detected in milk when the BLV PVL in blood was more than 10,000 copies/10^5^ cells [19]. However, in susceptible dams, BLV provirus was detected in milk in some cases and not in others during the follow-up study. One possible reason for this discrepancy may be the BLV PVL in blood. The present results demonstrated that the BLV PVL in milk was positively associated with PVL in peripheral blood and PVL values in milk were 18- to 270-fold lower than those measured in blood. In addition, our previous study reported partial sequence analysis results of BLV *env* confirmed that the BLV strains in milk samples were identical to those in matched blood samples from the three cows analyzed [19]. These findings support that BLV-infected cells are derived from peripheral blood and circulate throughout the body to be distributed in the mammary glands, including in milk. Since BLV-infected cells in milk are derived from blood cells, BLV provirus is detected in milk as the abundance of BLV-infected cells in blood increases [19]. The second possible reason for the discrepancy among susceptible dams can be attributed to the presence of numerous somatic cells in milk. Cattle infected with BLV reportedly experience a high incidence of mastitis [8,9]. Moreover, the average somatic cell count of some BLV-infected dams in this study was more than twice that of non-BLV-infected dams raised on the same farm (data not shown). Milk may contain various somatic cells, such as white blood and epithelial cells, and previous studies have suggested that BLV has a wide host range and can infect various cell types in vitro and ex vivo [42,43,44]. Therefore, the frequency of BLV provirus detection in milk may increase when dams have a high number of somatic cells in their milk.

Despite being classified as a resistant dam, milk samples from R1 tested positive for BLV provirus two of four times during the follow-up study, and BLV PVL in blood from R1 was higher than that in other resistant dams. In addition, we previously reported that R1 formed more than 10 times the number of syncytia than the blood of other resistant dams [26]. These results suggest that this dam may not effectively suppress BLV infection, even though the dam harbored a resistant allele. Resistance and susceptible alleles of Japanese Black cattle differ in terms of polymorphisms in DNA, amino acids, and binding pocket properties. Nevertheless, the structural differences between susceptible and resistant alleles of Holstein-Friesian cattle have not been examined [27]. Further analyses of susceptibility and resistance alleles in Holstein-Friesian cattle may reveal the mechanism by which resistant alleles fail to suppress BLV infection.

Results of the improved LuSIA revealed that milk samples of individuals with high PVL tended to be positive for infectivity. Likewise, we previously demonstrated the infectious capacity of milk cells when the PVL in milk was at least 30 copies/10^5^ cells. In addition, a previous three-year follow-up study with blood of BLV-infected cows revealed that the number of syncytia measured using LuSIA was positively correlated with the PVL in blood measured using the BLV-CoCoMo-qPCR-2 assay [26]. Taken together, these results suggest that the infectivity of milk cells might be associated with the PVL in milk and blood. However, one in vitro study reported that the antibodies in milk and colostrum of BLV-positive dams may protect against BLV infection [45]. Therefore, the effect of neutralizing antibodies on the infectivity of milk cells should be examined using our improved LuSIA in the near future. Nevertheless, it has been previously reported that BLV-infected cells are present in the milk and colostrum of BLV-positive dams [20], supporting the notion that milk is a potential risk factor for BLV spread through vertical transmission.

This study found that milk cells from susceptible BLV-infected dams were infectious, in contrast to those from resistant dams infected with BLV, further supporting that milk from infected susceptible cattle may be a source of vertical BLV transmission. Therefore, feeding milk to calves from BLV-infected susceptible but not BLV-infected resistant dams may be one of the most critical infectious routes of vertical transmission on dairy farms. In addition to milk, blood from susceptible cattle is highly infectious, whereas blood from resistant cattle is less infectious [26]. Taken together, these results indicate that the risk of vertical and horizontal transmission is extremely high for cattle harboring susceptible alleles compared to those carrying resistant alleles. The present study results will enable effective cattle-management policies for the control and eradication of BLV. For example, separating dams and calves as early as possible after calving may prevent vertical transmission of BLV through colostrum and milk. Notably, it is not easy for farmers to be present at every calving on dairy farms. Calves can then be fed freeze-treated colostrum or colostrum replacers to obtain transferable antibodies without infection with BLV. In particular, it is important to use milk from dams that have not been infected with BLV. Our study suggests that vertical milk-borne transmission on dairy farms can be reduced if farmers are present at the calving of susceptible cattle and perform early separation of dams and calves. This strategy provides a new BLV control measure that dairy farmers can easily implement. Although resistant cattle are at risk for vertical transmission, the risk is particularly low compared to that in susceptible cattle. Therefore, selective breeding of cattle with BLV-resistant *BoLA-DRB3* alleles rather than BLV-susceptible alleles could also reduce the risk of horizontal and vertical transmission and help develop an economically feasible and effective BLV eradication program under field conditions.

## 4. Materials and Methods

### 4.1. Cattle, Collection of Blood and Milk Samples, and Extraction of Genomic DNA

From 2017 to 2021, peripheral blood was collected from 672 Holstein-Friesian cattle from eight farms in Gunma, Tochigi, Saitama, Kanagawa, and Chiba prefectures in Japan (Table 1). BLV infection was determined using the BLV-CoCoMo-qPCR-2 assay (RIKEN Genesis), as previously described [34,35,36]. Farm A used a free-barn cowshed for housing, while the remaining seven farms used a tethered breeding housing system. All farms were generally managed as follows: (i) by using individual, single-use needles and syringes during vaccination or therapeutic protocols, (ii) by using individual, single-use obstetrical sleeves (or at least replaced between examination of BLV- infected and non-infected cattle), and (iii) by using disposable equipment (or at least cleaning, disinfecting, or sterilizing reusable materials and surgical instruments) in procedures such as dehorning, tattooing, implanting, cauterizing, castration, or ear-tagging. In addition, we sampled milk from 96 dams infected with BLV. Sampling of milk was carried out independently from the collection of blood samples for each individual. In total, 16, 4, 26, 9, 14, 14, 3, and 10 milk samples were collected from farms A, B, C, D, E, F, G, and H, respectively.

Genomic DNA was extracted from whole blood samples treated with ethylenediaminetetraacetic acid using the Wizard^®^ Genomic DNA Purification Kit (Promega Corporation, Madison, WI, USA), according to the manufacturer’s instructions. The genomic DNA concentration was measured using a Nanodrop 2000c spectrophotometer (Thermo Fisher Scientific, Waltham, MA, USA).

### 4.2. Separation of Milk Cells and Genomic DNA Extraction from Milk

Separation of milk cells and genomic DNA extraction were performed as previously described [19]. Briefly, 100 mL milk was collected from Holstein-Friesian cattle on each farm, maintained at 4 °C, and then transported to the laboratory within 24 h. Milk samples were transferred to 50-mL sterile tubes and centrifuged at 4000× *g* for 3 min to remove the cream layer, protein, and whey. The pellets, including milk cells and the remaining supernatant, were transferred to 15-mL sterile tubes and centrifuged at 1000× *g* for 30 min. The pellets were resuspended in 15 mL phosphate-buffered saline (PBS) and centrifuged twice at 1600 rpm for 5 min and 1200 rpm for 5 min.

DNA was extracted from milk cells using a Wizard Genomic DNA Purification Kit (Promega) with 1.54 mg/mL of dithiothreitol, according to the manufacturer’s instructions. The genomic DNA concentration in milk cells was measured using a Nanodrop 2000c spectrophotometer (Thermo Fisher Scientific, Waltham, MA, USA).

### 4.3. BLV PVL Detection

BLV PVL in blood and milk samples was determined using the BLV-CoCoMo-qPCR-2 assay [34,35,36] with THUNDERBIRD Probe qPCR Mix (Toyobo, Tokyo, Japan). Amplification was performed using the Light Cycler^®^ 480 System II (Roche Diagnostics, Mannheim, Germany) or the QuantStudio^®^ 5 Real-Time PCR System (Thermo Fisher Scientific). PVL was estimated as the copy number in 10^5^ white blood cells or milk cells.

### 4.4. BoLA-DRB3 Allele Typing

*BoLA-DRB3* was identified using the PCR-SBT method, as previously described [46]. Briefly, *BoLA-DRB3* exon 2 was amplified using PCR with DRB3FRW and DRB3 REV primers. The PCR fragments were purified and sequenced using the BigDye™ Terminator v1.1 Cycle Sequencing Kit (Thermo Fisher Scientific). Sequencing data were analyzed using ASSIGN 400 AFT software (Conexio Genomics, Fremantle, Australia) to determine *BoLA-DRB3* alleles.

### 4.5. Improved LuSIA

Milk cells were analyzed using an improved LuSIA [19,41,42]. Milk cells were resuspended in 1 mL Dulbecco’s modified Eagle’s medium (Thermo Fisher Scientific), supplemented with 10% fetal bovine serum (Sigma-Aldrich, St. Louis, MO, USA), and counted using a Neubauer chamber. Milk cells (1 × 10^5^ cells/well) were co-cultured with CC81-GREMG cells (1 × 10^4^ cells/well) and 1 µg/mL PWM in 12-well plate for 5 days. As a positive control, FLK-BLV cells permanently infected with BLV were co-cultured with CC81-GREMG cells, as previously described [41]. The cells were washed with PBS and subsequently fixed with PBS containing 3.7% formaldehyde and 10 mg/mL Hoechst 33342 (Sigma-Aldrich), as previously described [41]. Fluorescent EGFP-positive syncytia were observed in the fixed cells using an EVOS FL Auto 2 Imaging System (Thermo Fisher Scientific) or a BZ-X810 fluorescence microscope (Keyence, Osaka, Japan).

### 4.6. Statistical Analysis

R package version 4.1.2 was used to calculate *p*-values to determine the significance of differences between groups. The chi-square and Fisher’s exact tests were used to determine the significance of differences between the frequency of BLV provirus detection in milk of BLV-positive dams among susceptible, neutral, and resistant groups. *p*-values were adjusted using the Benjamini and Hochberg method. The Tukey’s multiple comparison test was used to determine the significance of differences in PVL in milk among susceptible, neutral, and resistant groups. *p*-value < 0.05 was considered statistically significant. The correlation coefficient (*r*) between PVL values in milk and blood was calculated using the Pearson function in Excel.

## 5. Conclusions

To the best of our knowledge, this is the first study to report an association between *BoLA-DRB3* polymorphism and BLV PVL in milk from Holstein-Friesian cattle infected with BLV. In this study, the BLV in milk from BLV-infected dams was analyzed transiently and continuously to determine the BLV provirus detection rate, PVL, and infectivity in milk. The following three main results were obtained. First, the detection rate of BLV provirus and BLV PVL in milk was significantly higher in susceptible dams than in resistant dams. Second, the tendency for milk from susceptible dams to have higher BLV provirus detection rates and BLV PVL than milk from resistant dams was found to persist over time. Third, milk cells from susceptible dams were infectious, whereas those from resistant cattle were non-infectious. Thus, *BoLA-DRB3* polymorphism controls the PVL and infectivity of milk from BLV-infected dams.

## Figures and Tables

**Table 1:** Sample size, bovine leukemia virus (BLV) prevalence, and distribution of susceptible, neutral, and resistant dams.

Farm ^a^	Total Cattle	BLV+ Cattle (%)	Dams					
			Total Dams	BLV+ (%) ^b^	Average PVL ^c^ in Blood	Susceptible ^d^ (%)	Neutral ^e^ (%)	Resistant ^f^ (%)
A	197	29 (14.7)	169	28 (16.6)	27,347	70 (41.4)	75 (44.4)	24 (14.2)
B	135	82 (60.7)	79	62 (78.5)	22,588	22 (27.9)	32 (40.5)	25 (31.6)
C	82	37 (45.1)	82	37 (45.1)	14,712	25 (30.5)	36 (43.9)	21 (25.6)
D	64	28 (43.8)	53	28 (52.8)	29,421	22 (41.5)	27 (50.9)	4 (7.6)
E	60	36 (60.0)	40	25 (62.5)	18,130	9 (22.5)	21 (52.5)	10 (25.0)
F	53	38 (71.7)	53	38 (71.7)	21,939	25 (47.2)	14 (26.4)	14 (26.4)
G	41	29 (70.7)	30	21 (73.3)	30,066	8 (26.7)	12 (40.0)	10 (33.3)
H	40	21 (52.5)	30	20 (52.5)	12,440	8 (26.7)	18 (60.0)	4 (13.3)
Total	672	300 (44.6)	536	259 (48.3)	22,080	189 (35.3)	235 (43.8)	112 (20.9)

^a^ In farms A, B, D, E, G and H, all dams, calves, and heifers were analyzed for BLV infection, but only dams were analyzed in farms C and F. ^b^ BLV+, BLV-positivity rate. ^c^ PVL: Proviral load (copies/10^5^ cells). BLV+ and PVL were determined using the BLV-CoCoMo-qPCR-2 assay. ^d,e,f^ *BoLA-DRB3* alleles were identified using PCR sequence-based typing. ^d^ Dams carried at least one susceptible BoLA-DRB3*012:01 or *015:01 allele but did not carry a resistant allele. ^e^ Dams did not carry susceptible or resistant alleles. ^f^ Dams carried at least one resistant BoLA-DRB3* 002:01, *009:02, or *014:01:01 allele.

**Table 2:** Longitudinal follow-up study of proviral load (PVL) in milk from susceptible and resistant dams.

Dam Classification	Dam	*BoLA-DRB3* ^c^		PVL in Milk / Blood (Copies/10^5^ Cells) ^d^						
				2017		2018			2019	
		A	B							
				Jul	Oct	Jan	May	Dec	Feb–Apr	Jun–Aug
Susceptible Dams ^a^	S1	012:01	015:01	N.T. ^e^	93 ^f^ / 63,280	N.T.	36 / 46,206	548 / 71,429	295 / 62,971	N.T.
	S2	011:01	015:01	159 / 49,799	4 / 37,484	74 / 38,038	N.T. / 45,215	N.T.	83 / 58,116	N.T. / 70,987
	S3	001:01	015:01	83 / 46,304	3 / 47,414	N.T. / 59,048	N.T. / 53,806	76 / 54,220	0 ^g^ / 74,644	N.T. / 76,296
	S4	011:01	015:01	N.T.	N.T. / 41,704	N.T.	42 / 24,824	41 / 37,059	0 / 27,260	0 / 18,904
	S5	011:01	015:01	N.T.	8 / 31,069	N.T.	0 / 26,739	N.T. / 35,838	77 / 32,016	66 /35,859
	S6	001:01	015:01	N.T.	N.T. / 24,621	N.T.	45 / 28,612	45 / 28,315	0 / 26,345	0 / 24,694
	S7	011:01	015:01	N.T.	N.T. / 24,500	N.T.	32 / 24,336	0 / 24,762	32 / 13,191	0 / 29,184
	S8	001:01	015:01	0 / 23,010	6 / 25,538	26 / 21,289	N.T. / 29,583	N.T. / 33,202	0 / 35,632	44 / 27,619
	S9	011:01	015:01	N.T.	0 / 22,331	N.T.	168 / 33,423	65 / 60,571	101 / 45,333	92 / 41,887
	S10	018:01	015:01	N.T.	N.T. / 20,561	0 / 20,976	N.T. / 30,497	N.T. / 50,333	226 / 53,039	123 / 65,556
	S11	010:01	015:01	N.T.	0 / 12,097	N.T.	N.T. / 25,179	46 / 42,888	24 / 30,878	0 / 23,826
	S12	010:01	015:01	76 / 11,545	54 / 10,557	N.T. / 7692	0 / 10,025	N.T.	N.T. / 15,323	N.T. / 11,397
	S13	011:01	015:01	N.T.	0 / 6389	N.T.	N.T. / 3529	0 / 10,663	0 / 8503	0 / 13,826
Resistant Dams ^b^	R1	011:01	014:01:01	N.T. / 12,251	N.T. / 15,581	0 / 15,190	108 / 13,260	N.T. / 17,337	102 / 20,380	0 / 28,531
	R2	015:01	009:02	0 / 168	N.T. /277	0 / 71	N.T. / 110	N.T. / 222	N.T. / 686	0 / 309
	R3	001:01	009:02	0 / 54	0 / 81	0 / 84	0 / 51	N.T. / 216	N.T.	0 / 188
	R4	014:01:01	014:01:01	0 / 32	0 / 63	0 / 0	N.T. / 909	0 / 0	0 / 0	0 / 114
	R5	011:01	014:01:01	N.T. / 0	N.T. / 0	N.T. / 2723	N.T. / 2645	N.T. / 3834	0 / 4871	0 / 2228

^a^ Susceptible dams carried at least one susceptible BoLA-DRB3*012:01 or *015:01 allele (red) but did not carry a resistant allele. ^b^ Resistant dams carried at least one resistant BoLA-DRB3*002:01, *009:02, or *014:01:01 allele (blue). ^c^ *BoLA-DRB3* alleles were identified using PCR sequence-based typing. ^d^ BLV PVL in milk (pink)/blood (black) were determined using the BLV-CoCoMo-qPCR-2 assay. ^e^ N.T., indicates the sample was not tested. ^f^ Orange shading indicates BLV provirus-positive milk. ^g^ 0 indicates that BLV provirus was not detected. BLV, bovine leukemia virus; PVL, proviral load.

**Table 3:** Results of improved luminescence syncytium induction assay (LuSIA) using milk cells from susceptible and resistant dams.

Dam Classification	Dam No.	*BoLA-DRB3* Allele ^c^		PVL (Copies/10^5^ Cells) ^d^		LuSIA in Milk
		A	B	Blood	Milk	
Susceptible Dams ^a^	S14	010:01	015:01	11,575	107	+ ^e^
	S2	011:01	015:01	49,799	70	+
	S15	011:01	015:01	35,859	66	+
	S16	001:01	015:01	24,696	56	+
	S17	027:03	012:01	72,014	54	- ^f^
	S18	027:03	012:01	84,479	47	-
	S19	011:01	015:01	578	32	-
	S20	011:01	012:01	53,985	30	+
	S21	027:03	012:01	58,734	0	-
Resistant Dams ^b^	R6	011:01	014:01:01	21,677	108	-
	R7	010:01	014:01:01	4710	51	-
	R8	011:01	002:01	3078	0	-
	R9	015:01	009:02	2413	0	-
	R10	014:01:01	014:01:01	112	0	-
	R11	001:01	009:02	27	0	-

^a^ Susceptible dams carried at least one susceptible *BoLA-DRB3* *012:01 or *015:01 allele (red) but did not carry resistant alleles. ^b^ Resistant dams carried at least one resistant BoLA-DRB3*002:01, *009:02, or *014:01:01 allele (blue). ^c^ *BoLA-DRB3* alleles were identified using PCR sequence-based typing. ^d^ BLV PVL was determined using the BLV-CoCoMo-qPCR-2 assay. ^e^ + indicates a positive result with improved LuSIA. ^f^ - indicates a negative result with improved LuSIA. BLV, bovine leukemia virus; PVL, proviral load.

## Data Availability

The data presented in this study are available upon request from the corresponding author.
